# Cystadenocarcinoma of the Sublingual Gland: A Rare Subvariant of Salivary Gland Carcinoma

**DOI:** 10.7759/cureus.69815

**Published:** 2024-09-20

**Authors:** Yuta Yasui, Kimio Uchiyama, Manabu Yamada, Kenichiro Suga, Seiji Asoda

**Affiliations:** 1 Department of Dentistry and Oral Surgery, National Hospital Organization (NHO) Tochigi Medical Center, Tochigi, JPN; 2 Department of Dentistry, Dental Clinic Shika-Nakahashi, Tokyo, JPN; 3 Department of Dentistry and Oral Surgery, Keio University School of Medicine, Tokyo, JPN

**Keywords:** cystadenocarcinoma, oral, salivary gland, salivary gland tumor, sublingual gland

## Abstract

Cystadenocarcinoma is a malignant tumor that has undergone various classifications due to its wide variety of pathological forms since the World Health Organization (WHO) classification in 2005. We present a case involving a 72-year-old man who reported pain and swelling in the left floor of his mouth during eating. Examination demonstrated a thumb-sized, mobile mass with elastic softness. Resection of the sublingual gland tumor and left submandibular neck dissection were performed under general anesthesia. The lesion was encased in a thin fibrous capsule and consisted of tall columnar epithelium with papillary proliferation of homogeneous eosinophilic columnar cells with minimal pleomorphism. It demonstrated continuous proliferation from the sublingual gland, extracapsular invasion, infiltrative growth, and mild nuclear atypia, leading to a diagnosis of cystadenocarcinoma. Owing to multiple lymph node metastases, left radical neck dissection was performed 21 months post surgery. The patient remains disease-free 85 months after surgery.

## Introduction

In the 2005 World Health Organization (WHO) classification [1], cystadenocarcinoma was designated as a malignant type of cystadenoma and as a subvariant of adenocarcinoma in the fourth edition of the WHO classification in 2017 [2]. It was subsequently classified as salivary gland carcinoma not otherwise specified (NOS) in the fifth edition in 2024 [3]. Typically, it features cyst formation and papillary proliferation into the cyst cavity [2]. The incidence of cystadenocarcinoma is low, comprising 0.18%-0.2% of salivary gland tumors [1]. Most cases occur in the parotid gland [4], with occurrences in the sublingual gland being rare. The sublingual gland is the third largest salivary gland and is primarily responsible for producing mucous saliva. It is located on the floor of the mouth between the tongue and the mylohyoid muscle. This tumor generally exhibits slow growth as an asymptomatic mass, with local recurrence and metastasis being uncommon, leading to a relatively favorable prognosis. Nonetheless, recurrence and metastasis can occasionally occur [5].

Here, we aimed to report a rare case of cystadenocarcinoma arising in the sublingual gland, together with a literature review.

## Case presentation

**Figure 1:**
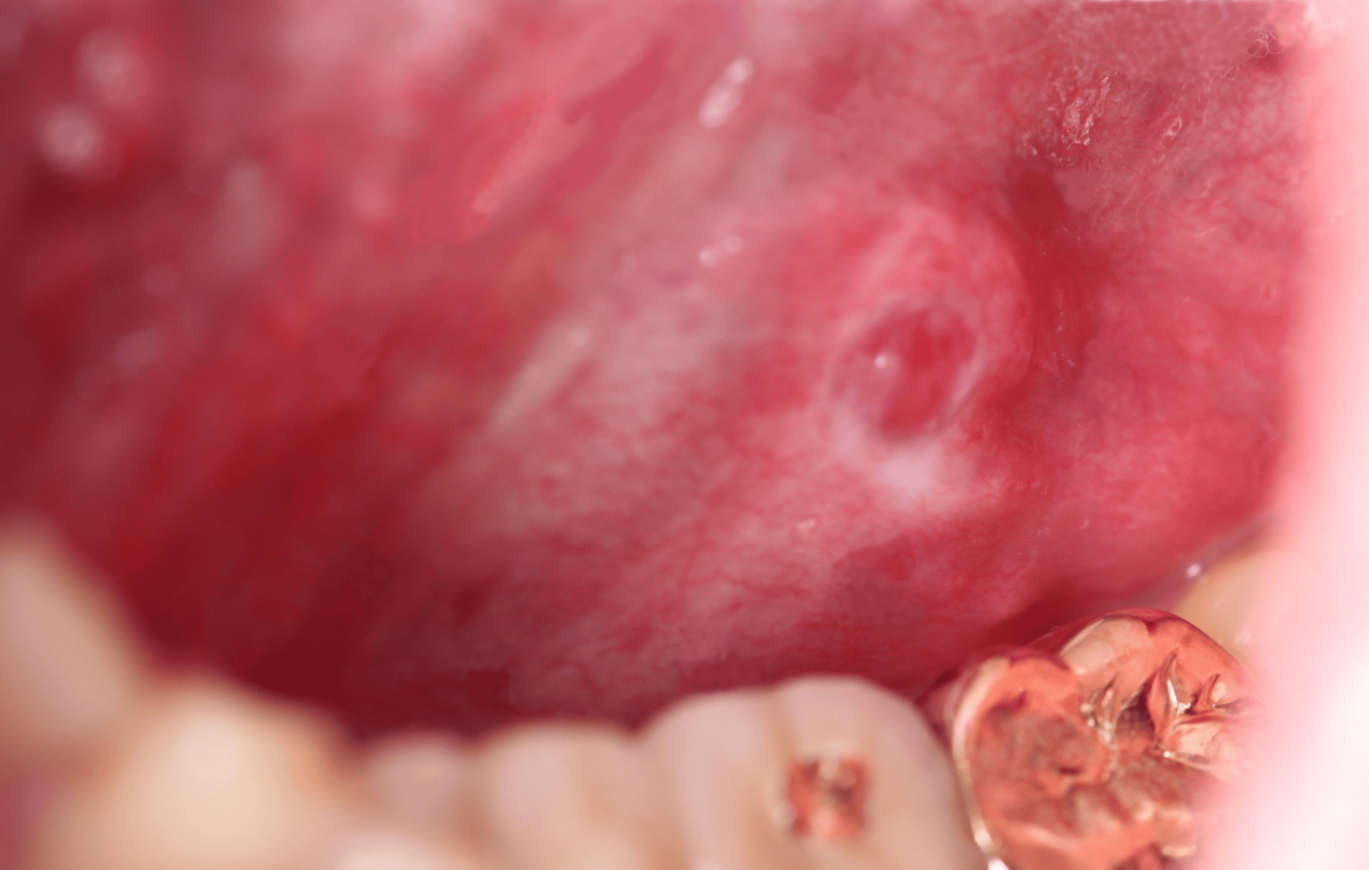
Intraoral findings during the initial examination A thumb-sized mass with a smooth mucosal surface and slightly unclear borders was observed on the left floor of the mouth.

**Figure 2:**
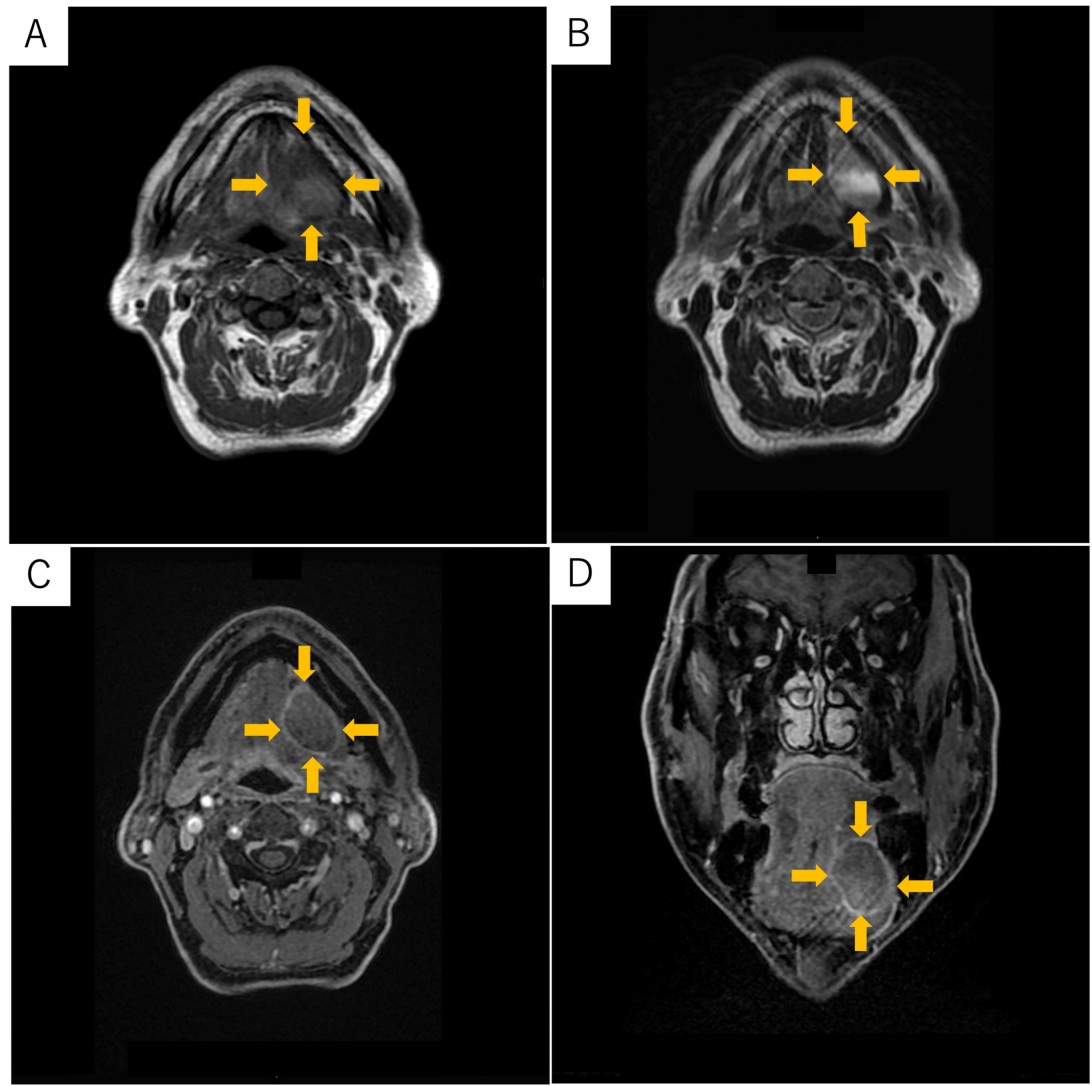
Magnetic resonance imaging reports (A) T1-weighted image (horizontal plane); (B) T2-weighted image (horizontal plane); (C) Gadolinium contrast T1-weighted image (horizontal plane); (D) Gadolinium contrast T1-weighted image (coronal plane). A 31-mm-diameter tumorous lesion on the left floor of the mouth was seen with low to medium signals on T1-weighted images and internally heterogeneous medium hyperintensity on T2-weighted images. The contrast-enhanced MRI demonstrated internally heterogeneous contrast effects.

In November 2014, a 72-year-old man sought further examination and treatment for a mass on the left floor of his mouth. He had experienced pain and swelling in the area while eating since October that year. His medical history included hypertension, complete right bundle branch block, and gout. Upon his initial visit, his facial features were symmetrical, and abnormal findings such as swelling or tenderness in the regional lymph nodes were not observed. Intraoral examination revealed a thumb-sized mass with a smooth mucosal surface and slightly unclear borders on the left floor of the mouth. There was no spontaneous pain, tenderness, or sensory abnormalities in the lingual nerve, but a slight trismus was present (Figure 1). Magnetic resonance imaging (MRI) indicated a 31-mm-diameter tumorous lesion in the left floor of the mouth, with low to medium signals on T1-weighted images and internally heterogeneous medium hyperintensity on T2-weighted images. The contrast-enhanced MRI demonstrated internally heterogeneous contrast effects (Figure 2). A biopsy was performed under local anesthesia, and the pathological diagnosis was a cystadenoma with a glandular to papillary structure formed by the proliferation of eosinophilic columnar epithelial cells with little atypia. F-fluorodeoxyglucose positron emission tomography/computed tomography (FDG-PET/CT) showed no significant accumulation in other organs, but strong accumulation (SUVmax: 4.5) was observed in the left sublingual gland area. Given that most sublingual gland tumors are malignant, an excision of the sublingual gland and left submandibular neck dissection were conducted under general anesthesia in July 2015. The tumor was resected en bloc, including the sublingual and submandibular glands, intrinsic tongue muscle, and mylohyoid muscle, along with some healthy surrounding tissue. No adhesions were observed between the mandible and the tumor. The resected material was elastically hard, covered with a membrane, and showed no internal fluid or necrotic areas. The defect was immediately reconstructed using a sternocleidomastoid myocutaneous flap.

**Figure 3:**
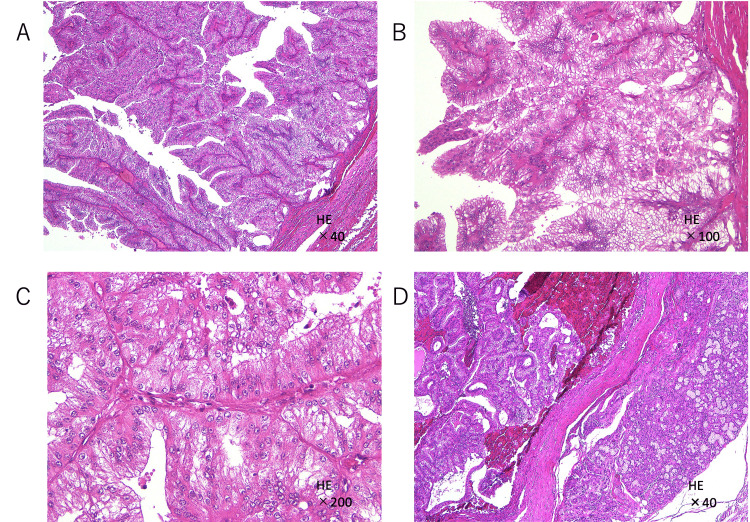
Histopathological findings (hematoxylin-eosin stain) (A, B, C) The lesion was encased in a thin fibrous capsule and lined with tall columnar epithelium exhibiting papillary proliferation of uniform eosinophilic columnar cells with minimal pleomorphism; (D) The tumor demonstrated continuous proliferation from the sublingual gland, with extracapsular invasion, infiltrative growth, and mild nuclear atypia.

Histopathological analysis revealed that the lesion was encased in a thin fibrous capsule and lined with tall columnar epithelium, exhibiting papillary proliferation of uniform eosinophilic columnar cells with minimal pleomorphism. The tumor demonstrated continuous proliferation from the sublingual gland, with extracapsular invasion, infiltrative growth, and mild nuclear atypia (Figure 3). Based on these findings, the lesion was ultimately diagnosed as cystadenocarcinoma (pT3N0M0, stageⅢ) originating from the sublingual gland.

**Figure 4:**
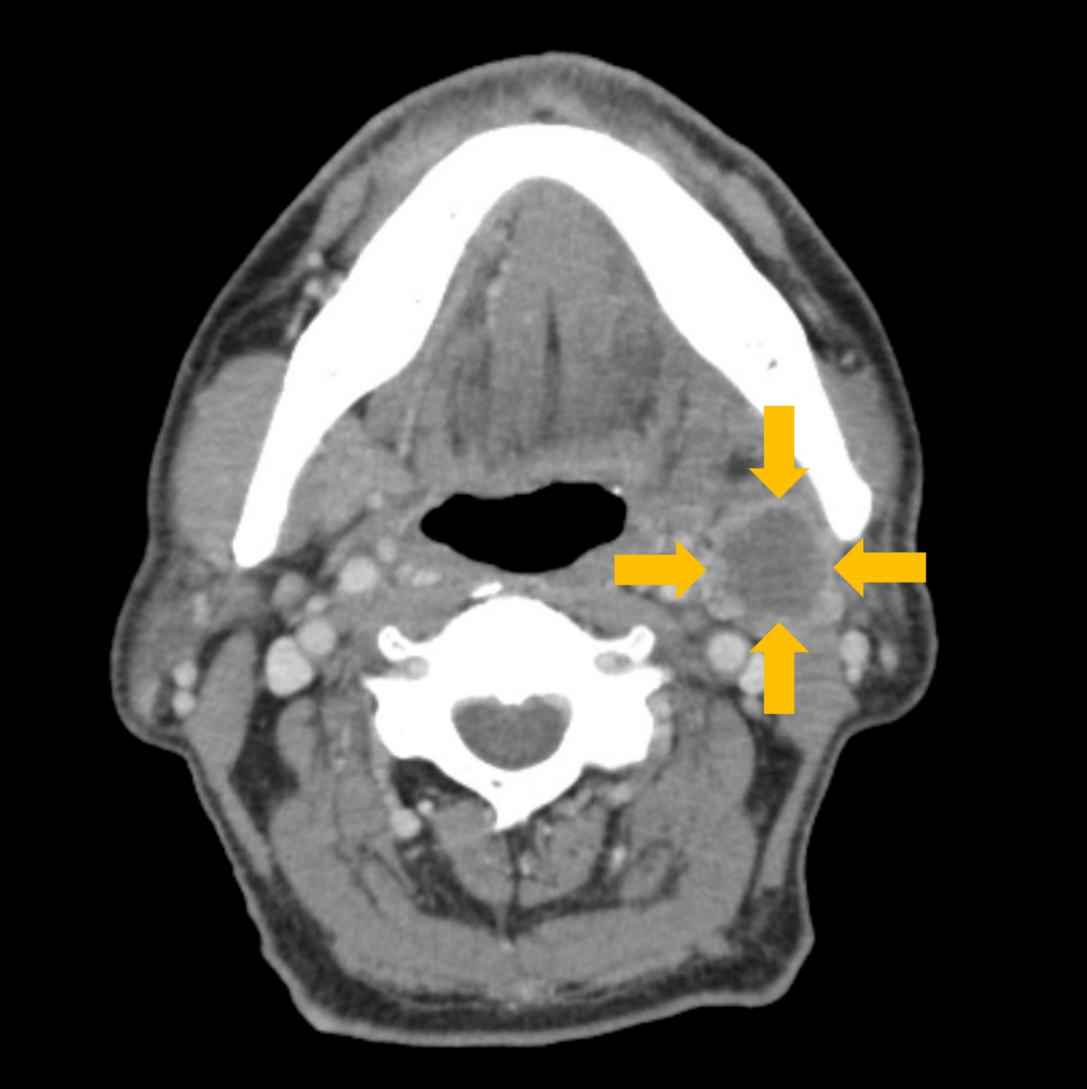
Contrast computed tomography image of 21 months post surgery (horizontal plane) Lymph node metastases on the left side of the neck were suspected.

A CT scan taken 21 months post surgery indicated multiple lymph node metastases on the left side of the neck (Figure 4). Consequently, a left radical neck dissection was performed under general anesthesia as an additional treatment. Pale to eosinophilic columnar cells forming a papillary structure were found in one superior deep cervical lymph node and two middle deep cervical lymph nodes, which were identified as metastases from the previous lesion. No extranodal extension was evident; thus, no further treatment was administered.

Currently, 85 months have elapsed since the additional treatment, and there has been no evidence of tumor recurrence or distant metastasis. The patient is satisfied with the treatment and is progressing well.

## Discussion

Cystadenocarcinoma shows no sex predilection and most commonly occurs in individuals over 50 years of age. It is most frequently found in the ovaries, gallbladder, pancreas, breast, thyroid, and upper respiratory tract. Although it can occur in the salivary glands of the oral and maxillofacial region, it is an extremely rare condition, representing 0.18%-0.2% of all salivary gland tumors [4]. Foss et al. [5] examined 57 cases of cystadenocarcinoma in the salivary glands and found it most frequently affects individuals over 50 years of age, with no sex predilection. Approximately 60% of cases occur in the parotid gland, 35% in minor salivary glands such as the lips, and only 3.5% in the sublingual gland. Our review identified six reported cases of cystadenocarcinoma in the sublingual gland, including our own case [6-10] (Table 1). The average age of these cases was 56.5 years, with two cases occurring in individuals in their 30s. There were more male patients than female, with five males and one female.

**Table 1:** Cystadenocarcinoma in the sublingual gland CLNs: cervical lymph nodes; NA: not available; NED: no evidence of disease; FNA: fine-needle aspiration; DOD: dead of disease; AWD: alive with disease

Authors	Year	Sex	Age	Size	Symptoms	Duration of illness	Biopsy method	Diagnosis at biopsy	Metastasis at presentation	Treatment	Cell type	Treatments in Addition to Surgery	Recurrence/metastasis	Time of recurrence	Treatment of recurrence	Follow-up
Danford et al. [6]	1992	M	38	NA	Swelling	2 weeks	FNA	Class Ⅰ (negative)	NA	Excision of the right sublingual gland and associated cyst	Tall columnar	Wide excision of the floor of the mouth and the right side of the tongue with a right supraomohyoid neck dissection	-	-	-	NED at 6 months
Kobayashi et al. [7]	1999	M	55	50×35×30mm	Swelling, tenderness	NA	NA	NA	No	A partial resection of the floor of the mouth and a suprahyoid neck dissection on the left side	Tall columnar	No	-	-	-	NED at 1 year
Yamada et al. [8]	2007	M	67	30×25×3mm	Mass in the right submandibular region and elastic hard and tender mass on the oral floor of the right molar region of the mandible	10 years	NA	NA	NA	Enucleation	Tall columnar	No	The mental region and the right cervical lymph nodes	3 months	Segmental resection of the mandible and total neck dissection→chemo therapy(TS-1+CDDP)	AWD at 18 months after additional treatment
Neil Giblett [9]	2017	M	68	55×60×30mm	Neck swelling in the left submandibular region, excessive sweating, and unexplained lethargy	6 months	FNA	Cystic lesion (negative)	No	Surgical excision was performed through the neck; neck dissection was not performed)	Admixed	Postoperative radiotherapy	NA	NA	NA	NA
Silva et al. [10]	2022	F	39	40mm	Swelling	3 months	FNA and incisional biopsy	Adenocarcinoma not otherwise specified	Level I: 3/4, level II: 3/4, and level III: 5/10	Hemimandibulectomy associated with left supraomohyoid neck dissection	NA	Adjuvant chemotherapy	Transition between the buccal mucosa and oropharynx	1 year	NA	DOD at 1.5 years
Present case	2014	M	72	31mm	Swelling, tenderness	1 month	Incisional biopsy	Cystadenoma	-	Wide local excision, upper neck dissection	Tall columnar	-	Left cervical lymph nodes	21 months after surgery	Left radical neck dissection	NED at 65 months after additional treatment

The WHO [11] and the Armed Forces Institute of Pathology (AFIP) [4] classify this tumor as a low-grade malignant tumor. Clinically, cystadenocarcinoma presents with few subjective symptoms, leading to a prolonged period from symptom onset to a hospital visit, which ranges from one month to five years, with an average of 14 months [5], an unusually long duration for a malignant tumor. It gradually grows and is often asymptomatic. Among the six cases mentioned, all patients experienced swelling, which led to the tumor’s discovery, and two cases reported pain. Consistent with previous reports, the average disease duration was 26 months, and tumors were discovered when they had grown relatively large, measuring between 30 mm and 60 mm. Furthermore, five cases, including this one [7-10], were depicted as lesions with relatively clear borders on imaging findings. Although cystadenocarcinoma is a malignant tumor, this distinct characteristic is frequently observed.

Histopathologically, cystadenocarcinoma is characterized by the formation of cystic cavities and papillary proliferation of atypical glandular epithelium within these cavities. The atypical glandular epithelium, ranging from cuboidal to columnar cells, forms cystic cavities of numerous sizes while infiltrating and proliferating into the surrounding tissues [1]. Foss et al. [5] categorized the lining epithelium of the cystic cavities into four types according to the shape of the tumor cells: small cuboidal cell type (61.4%), large cuboidal cell type (15.8%), tall columnar cell type (12.3%), and admixed cell type (10.5%). They reported that 75% of cases with metastasis were of the tall columnar type. Pollett et al. [12] also classified the tall columnar type as highly malignant due to the high mitotic activity, large nuclei, and pronounced atypia of the lining epithelium. Although cystadenocarcinoma is generally considered a low malignant type, four of the six cases (67%), including our own, were of the tall columnar type, with three demonstrating recurrence or metastasis after initial treatment. Thus, it was considered necessary to decide the treatment strategy for cystadenocarcinoma arising in the sublingual gland, considering its high malignancy and the possibility that it is prone to recurrence and metastasis.

Given the characteristics of the tumor, the differential diagnosis includes benign tumors such as cystadenoma, intraductal papilloma, and pleomorphic adenoma, as well as glandular carcinomas such as low-grade pleomorphic adenocarcinoma and mucoepidermoid carcinoma. Among the six cases we identified, two were examined via fine needle aspiration cytology (FNA), one by biopsy, and one by both FNA and biopsy. However, three out of four cases were initially diagnosed as benign. When the tumor occurs in the sublingual gland, the possibility of malignancy must always be considered [13]. In our case, the initial pathological diagnosis from the biopsy was a benign tumor, specifically a cystadenoma. However, PET/CT demonstrated strong FDG accumulation in the same area, and given that most sublingual gland tumors are malignant and the time from symptom onset to the first consultation was only one month, we proceeded with resection as if treating a malignant tumor.

Surgical resection remains the primary treatment for cystadenocarcinoma, similar to other salivary gland tumors, as it is generally considered to be of low malignancy [11]. The role of radiation therapy and chemotherapy as adjunct treatments has not been clearly established. However, Terhaard et al. reported that, among 498 cases of malignant salivary gland tumors, postoperative radiation therapy was beneficial in cases with positive or close resection margins, perineural invasion, or T3-T4 tumors. Postoperative radiation therapy significantly improved local control rates over a 10-year period compared to surgery alone (58% vs. 87%) [14]. Furthermore, in cases with extracapsular invasion or internal fluid leakage, recurrence was sometimes prevented by supplementary radiation therapy, additional resection, or neck dissection [15-18]. In the six identified cases, all underwent surgery, but only one was diagnosed preoperatively, and the surgical approaches varied. Foss et al. [5] explored 40 cases of primary salivary gland tumors and found local recurrence in three cases (7.5%), lymph node metastasis in three cases at initial presentation, and one case 55 months post-surgery, totaling four cases (10.0%). Among the six cases of sublingual gland cystadenocarcinoma reviewed, recurrence and metastasis were observed in three cases (50.0%), as noted. Therefore, although cystadenocarcinoma is considered a low-grade malignant tumor, tall columnar-type cystadenocarcinoma is considered a high-grade tumor even if it is covered by a membrane, and resection margins should be set at least 10 mm. After treatment, long-term follow-up observation once every few months for at least five years is necessary. In this case, the patient has been doing well with no evidence of recurrence or metastasis 85 months after additional treatment; however, further careful follow-up is warranted.

## Conclusions

We report a rare case of cystadenocarcinoma arising in the sublingual gland, highlighting the importance of accurate diagnosis and timely treatment. As demonstrated in this case, cystadenocarcinoma originating from the sublingual gland, especially the tall columnar type, is highly malignant and predisposed to recurrence and metastasis. Our case demonstrates the successful resection of the tumor but emphasizes the need for careful consideration of the treatment plan, as reported in the literature.
